# Oral Microbiome as a Biomarker and Therapeutic Target in Head and Neck Cancer: Current Insights and Future Directions

**DOI:** 10.3390/cancers17162667

**Published:** 2025-08-15

**Authors:** Saad Ahmad, Dasantha Jayamanne, Sarah Bergamin, Anna Lawless, Alexander Guminski, Adrian Lee, Alexander Yuile, Helen Wheeler, Thomas Eade, Michael Back, Mark Molloy, Byeongsang Oh

**Affiliations:** 1 Faculty of Medicine and Health, University of Sydney, Sydney, NSW 2006, Australia; hafizsaad.ahmad@sydney.edu.au (S.A.);; 2 Northern Sydney Cancer Centre, Royal North Shore Hospital, St Leonards, NSW 2065, Australia; 3 Bowel Cancer and Biomarker Laboratory, Kolling Institute, St Leonards, NSW 2065, Australia; 4 Bill Walsh Translational Cancer Research Laboratory, Kolling Institute, St Leonards, NSW 2065, Australia

**Keywords:** oral microbiome, head and neck cancer, cancer biomarker, microbiome dysbiosis, oral squamous cell carcinoma

## Abstract

**Background/Objectives**: The oral microbiome has been implicated in the pathogenesis of head and neck squamous cell carcinoma (HNSCC). This review examines the association between specific oral bacterial taxa and HNSCC. **Methods**: A systematic review was conducted following the Preferred Reporting Items for Systematic Reviews and Meta-Analyses (PRISMA) guidelines to examine the relationship between the oral microbiome and HNSCC. A comprehensive literature search was conducted in databases including EMBASE, Cochrane Library, Web of Science, Medline, and PubMed. **Results**: Following the screening of 284 articles, 21 studies met the inclusion criteria, comprising 1023 HNSCC patients (male: n = 806, 79%; female: n = 217, 21%) and 837 healthy controls (male: n = 622, 74%; female: n = 215, 25.7%). Although findings on alpha diversity were inconsistent, a significant difference in beta diversity was consistently reported between HNSCC patients and healthy controls. HNSCC patients exhibited higher relative abundances of *Firmicutes* and *Synergistetes* at the phylum level; *Fusobacterium*, *Prevotella*, *Porphyromonas*, *Parvimonas*, and *Peptostreptococcus* at the genus level; and *Fusobacterium nucleatum*, *Prevotella intermedia*, *Lactobacillus* spp., and *Porphyromonas gingivalis* at the species level. In contrast, healthy controls showed higher abundances of *Proteobacteria* and *Actinobacteria* at the phylum level; *Streptococcus*, *Actinomyces*, *Corynebacterium*, *Rothia*, and *Veillonella* at the genus level; and *Haemophilus influenzae*, *Rothia mucilaginosa*, and *Streptococcus mitis* at the species level in most studies. **Conclusions**: The findings indicate distinct alterations in oral microbiome diversity and composition among HNSCC patients, highlighting the role of microbial dysbiosis in cancer progression. Standardized protocols for oral sample collection and microbiota analysis are essential to facilitate more robust, comparable, and clinically meaningful research outcomes.

## Simple Summary

Emerging evidence suggests that the oral microbiome—the community of microorganisms in the mouth—may influence the development of head and neck squamous cell carcinoma (HNSCC), a common type of cancer. This review summarizes current research comparing the oral microbiome of HNSCC patients with that of healthy individuals. Distinct differences in bacterial composition were observed, with certain species being more prevalent in cancer patients. These microbial shifts may be associated with inflammation and other cancer-promoting mechanisms. A better understanding of this microbiome changes could improve early risk identification and inform new strategies for prevention or treatment.

## 1. Introduction

Head and neck squamous cell carcinomas (HNSCCs), arising from the mucosal epithelium of the oral cavity, pharynx, and larynx, represent the most common malignancies in the head and neck region. The global prevalence of HNSCC varies across regions and is primarily attributed to exposure to tobacco-derived carcinogens, excessive alcohol consumption, or both. Further, infection with oncogenic strains of human papillomavirus (HPV), particularly in the oropharynx, has emerged as a significant etiological factor [1,2]. In 2020, HNSCC was the eighth most common cancer globally, with an estimated 878,000 new cases and 442,000 deaths. By 2030, the annual incidence is projected to increase by 30%, reaching an estimated 1.08 million new cases [3]. Standard treatment modalities for HNSCC include surgical resection, radiotherapy (with or without chemotherapy), or a combination of both, depending on disease location and stage [4]. The estimated 5-year survival rate in the USA ranges from 87% for cases diagnosed at a localized stage to 40% for those diagnosed with metastatic disease; however, these rates vary significantly by geographic region [5]. This significant global disease burden highlights the pressing need for novel strategies in early diagnosis, prognosis, and treatment. Emerging evidence over the past two decades suggests that alterations in the human microbiota may disrupt the host–microbe balance, potentially contributing to carcinogenesis alongside established risk factors [6]. Bacteria have been implicated in cancer development through mechanisms such as chronic inflammation, inhibition of apoptosis, and production of carcinogenic metabolites [7]. Advances in omics technologies, including metagenomics, transcriptomics, and proteomics, have enabled deeper insights into the microbiome’s role in various diseases, including HNSCC [8]. Established links between specific bacterial species and cancer types, for example, *Helicobacter pylori* in gastric cancer, *Salmonella enterica* in colorectal cancer, and *Salmonella Typhi* in gallbladder cancer, support the plausibility of microbial involvement in tumorigenesis [9]. Multiple studies have used next-generation sequencing (NGS) to characterize the oral microbiota in HNSCC patients, aiming to identify specific microbial signatures associated with the disease and determine whether microbial dysbiosis is a cause or consequence of carcinogenesis [6,10]. In addition, evidence from preclinical studies suggests that *Fusobacterium nucleatum* and *Porphyromonas gingivalis* contribute to cancer development through pro-inflammatory and immunomodulatory mechanisms [11,12]. *F. nucleatum* promotes tumor progression by inducing cytokines (e.g., TNF-α, IL-6, IL-8), enhancing cell proliferation, inhibiting apoptosis, and recruiting immunosuppressive cells [13]. *P. gingivalis*, a key pathogen in periodontitis, similarly stimulates inflammatory cytokines and supports tumor cell survival, angiogenesis, and immune evasion [14]. Both bacteria can disrupt host immune responses and may act synergistically to amplify inflammation and tumor-promoting pathways [15]. Although these findings are primarily derived from preclinical models, they provide a biologically plausible link between oral dysbiosis and carcinogenesis.

While microbial alterations have been observed, inconsistencies across clinical studies highlight the need for a comprehensive synthesis of the current evidence. This review examines recent literature investigating the relationship between the oral microbiome and HNSCC. By consolidating available data and accounting for discrepancies, this study aims to assess the potential of the oral microbiome as a biomarker for early detection and as a therapeutic target in the management of HNSCC.

## 2. Materials and Methods

**Figure 1:**
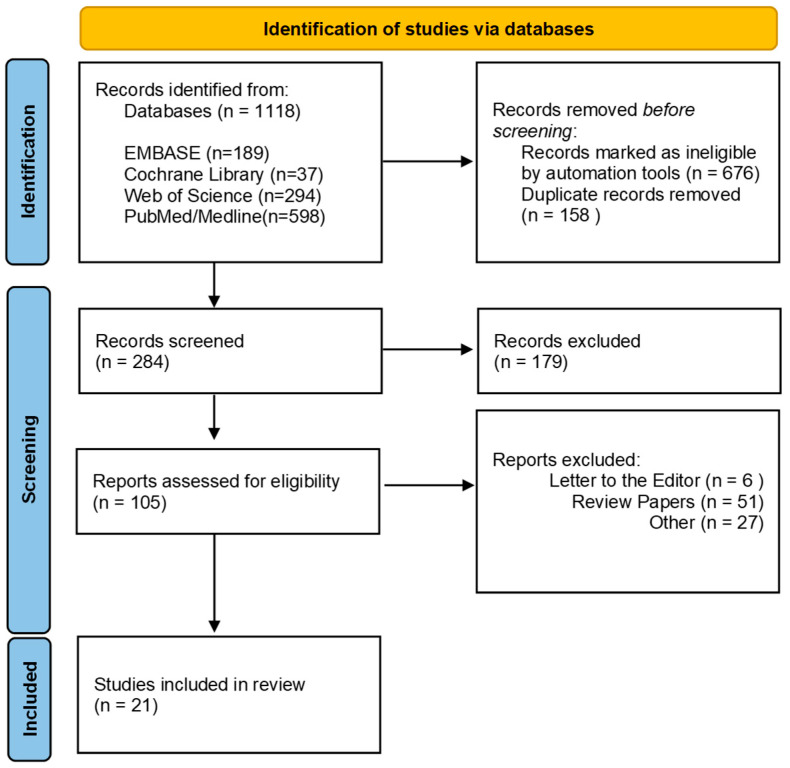
PRISMA flow diagram for systematic analysis.

This narrative review was conducted in accordance with the Preferred Reporting Items for Systematic Reviews and Meta-Analyses (PRISMA) guidelines. A comprehensive literature search was performed to identify relevant studies examining differences in the oral microbiome between patients with HNSCC and healthy controls. The search covered four electronic databases—EMBASE, Cochrane Library, Web of Science and PubMed/Medline (Figure 1)—and included publications from December 2014 to December 2024.

The primary research question guiding the review was as follows: *Are there differences in the composition of the oral microbiota between patients with HNSCC and healthy individuals?* The search strategy included the following keywords and phrases: “head and neck squamous cell carcinoma,” “oral cancer and microbiome,” “oral microbiome,” “oral microbiota,” and combinations thereof using Boolean operators “AND” and “OR” to refine the results.

Inclusion criteria were as follows: (1) original research articles published in English between 2014 and 2024; (2) full-text availability; and (3) studies that compared the oral microbiome of HNSCC patients with that of healthy individuals or paired para-cancerous tissues. Articles were initially screened by title and abstract, followed by full-text assessment for eligibility.

Studies were excluded if they met any of the following criteria: (1) non-original research (e.g., reviews, commentaries, case reports, editorials, or conference abstracts); (2) studies without a direct comparison between HNSCC/OSCC patients and healthy controls; (3) in vitro or animal studies; and (4) articles not available in English or lacking full-text access. This is a narrative review rather than a systematic review; thus, it was not registered in databases such as PROSPERO. This review aims to provide a timely synthesis of emerging evidence for researchers, clinicians, and the general public, with a focus on new insights and evolving trends in head and neck cancer.

## 3. Results

### 3.1. Characteristics of Studies

A total of 21 studies from December 2014 to December 2024 met the inclusion criteria and were included in this review (see Figure 1). The initial literature search yielded 1118 records. After screening according to the PRISMA guidelines, 105 articles were assessed for eligibility, and 21 studies were included. The studies spanned a broad geographical distribution, including North America (USA, n = 8) [16,17,18,19,20,21,22,23], Asia (China: n = 4, India: n = 2, Taiwan: n= 1) [6,24,25,26,27,28,29], Europe (Finland: n = 1, Turkey: n = 1) [30,31], South America (Brazil, n = 1) [32], and Oceania (Australia: n = 2, New Zealand: n = 1) [33,34,35]. Across the included studies, the average age of participants ranged from 58 to 60 years. No significant demographic differences were reported between case and control groups regarding age, body mass index (BMI), gender, smoking history, or alcohol consumption. In total, the studies comprised 1023 HNSCC patients (males, n = 806 and females, n = 217) and 837 healthy controls (males, n = 622 and females, n = 215). The number of participants per study ranged from 10 to 129 in the patient group, and from 7 to 254 in the control group. Of 21 studies, 17 were case–control studies comparing the oral microbiome of HNSCC patients with healthy individuals, and 4 were paired tissue studies comparing cancerous and adjacent non-cancerous tissues. Sample types analysed included oral rinse/wash (n = 7) [6,17,19,20,21,25,33], saliva (n = 5) [20,24,28,30,31], tissue samples (n = 3) [23,26,29], oral swabs (n = 2) [18,32], and oral mucosa brushing (n = 1) [16]. Further, three studies employed multiple sample types: saliva, tissue, and oral swabs [34]; saliva and oral swabs [22]; and tissue, saliva, and mouthwash [27]. Regarding sequencing approaches, 18 studies employed 16S ribosomal RNA (rRNA) gene sequencing, and 3 used metagenomic shotgun sequencing [20,21,24]. Among those using 16S rRNA sequencing, the most frequently targeted hypervariable regions were V3–V4 (n = 10) [6,19,22,25,26,28,29,31,34,35], followed by V4 (n = 3) [16,17,30], V1–V2 (n = 2) [27,32], V1–V4 (n = 1) [23], and V6–V8 (n = 1) [33] (see Table 1).

Alpha diversity (diversity within a single habitat) was evaluated in 20 of 21 studies. Three studies reported significantly higher alpha diversity in HNSCC patients than in controls [6,33,35]. Seven studies significantly lower alpha diversity [16,25,27,30,31,32,34], and ten studies found no significant difference.

Beta diversity, (diversity between habitats) was assessed in all 21 studies. Sixteen studies found significant differences in beta diversity between HNSCC patients and control groups, four studies reported no significant differences in the oral microbiota composition between HNSCC patients and healthy controls [20,26,29,32], and one study reported higher beta diversity in the patient group [17].

### 3.2. Oral Microbiome in HNSCC

#### 3.2.1. Phylum-Level Differences Between HNSCC and Controls

Of the 21 studies included, 10 reported significant differences in the relative abundance of bacterial phyla between patients with HNSCC and healthy controls [6,21,23,24,26,27,28,29,31,33]. The predominant phyla identified were *Firmicutes*, *Actinobacteria*, *Proteobacteria*, and *Synergistetes*. *Firmicutes* was the most frequently reported phylum, identified in both HNSCC and control groups across eight studies; five studies reported higher abundance in HNSCC patients [26,28,29,31,33], while three reported higher abundance in controls [21,23,27]. *Actinobacteria* was less abundant in HNSCC samples in five studies [21,24,27,28,29], with no studies reporting reduced abundance in controls. *Proteobacteria* was reported to be less abundant in patients in three studies [28,29,31]; one study noted a lower abundance in controls [27], though statistical significance was not consistently reported. *Synergistetes* was reported in only one study [21] where it was elevated in HNSCC patients compared to controls (see Table 2).

#### 3.2.2. Class, Order and Family Level Differences

Seven studies reported differences in bacterial abundance at the class level [6,21,26,27,28,29,33]. *Bacteroidetes* and *Fusobacteria* were more abundant in HNSCC groups across all seven studies [6,21,26,27,28,29,33] that investigated these taxa. Only one study presented findings at the order level [28] and another reported data at the family level [17] limiting generalization at these taxonomic ranks.

#### 3.2.3. Genus-Level Differences

Nineteen studies reported genus-level differences in the oral microbiota between HNSCC patients and healthy controls. The most frequently identified genera among them were *Fusobacterium* (n = 14) [6,17,18,21,24,25,26,27,29,31,32,33,34,35], *Streptococcus* (n = 12) [6,18,21,24,25,26,27,29,31,32,33,34], *Actinomyces* (n = 7) [6,18,21,23,30,32,35], *Prevotella* (n = 6) [26,29,30,32,33,34], *Corynebacterium* (n = 6) [19,20,21,24,32,34], *Porphyromonas* (n = 5) [6,26,32,33,34], *Parvimonas* (n = 5) [6,18,23,25,29], and *Rothia* (n = 5) [18,25,26,27,32]. *Fusobacterium* was consistently elevated in HNSCC samples. Conversely, *Streptococcus*, *Actinomyces,* and *Rothia* were more frequently associated with control samples. In contrast, *Prevotella, Porphyromonas*, and *Parvimonas* were predominantly elevated in HNSCC groups. Several genera showed inconsistent trends. *Leptotrichia* was more abundant in HNSCC patients in two studies [25,29] and in controls in two others [28,32]. *Peptostreptococcus* (n = 4) was more prevalent in HNSCC samples [18,25,29,31], while *Veillonella* (n = 4) was consistently less abundant in this group [24,26,30,34]. *Two studies each reported that Campylobacter* [27,28], *Capnocytophaga* [25,29], *Gemella* [24,31], and *Treponema* [18,35] were more abundant in the HNSCC group, while *Neisseria* [28,33], and *Haemophilus* were abundant in the control [6,29].

Several genera were identified in only one study, including *Bacillus* [17], *Eikenella* [17], *Granulicatella* [24], *Lachnospira* [24], *Megasphaera* [18], *Kingella* [19], and *Selenomonas* [21].

#### 3.2.4. Species-Level Differences

In total, 10 of the 21 studies provided species-level resolution. *Fusobacterium nucleatum* was the most frequently identified species, reported in seven studies [16,20,26,27,29,30,31], and was increased in HNSCC patients in all seven. *Prevotella intermedia* was reported in four studies [16,20,26,29] to be consistently elevated in HNSCC patients. Other species appearing in at least two studies included *Haemophilus influenzae*, *Haemophilus parainfluenzae*, *Lactobacillus* spp., *Porphyromonas gingivalis*, *Rothia mucilaginosa*, and *Streptococcus mitis*. *Haemophilus influenzae* showed variable trends: one study reported increased abundance in HNSCC patients, while two studies reported higher levels in controls. Both *Porphyromonas gingivalis* and *Lactobacillus* spp. were elevated in HNSCC samples [16,20,31]. In contrast, *Rothia mucilaginosa* and *Streptococcus mitis* were more frequently identified in control groups [22,27,29].

## 4. Discussion

This review highlights significant alterations in microbial diversity and composition between HNSCC patients and healthy controls, consistent with previous studies [36,37,38] supporting the hypothesis that microbial dysbiosis may contribute to carcinogenesis [39]. However, the differences varied widely between studies. Among the 20 studies assessing alpha diversity, 3 reported statistically significant reductions in HNSCC patients [6,33,35], 7 observed a decreasing trend [16,25,27,30,31,32,34], and 10 found no significant differences [17,18,19,20,21,23,24,26,28,29]. With regard to beta diversity, 16 of 21 studies reported significant compositional differences between HNSCC patients and healthy individuals. While these findings suggest a potential association between microbial diversity and HNSCC [40], the inconsistencies across studies underscore the need for standardized methodologies and larger, well-characterized cohorts to clarify this relationship.

Oral Microbiota Alterations in HNSCC Patients: At the phylum level, *Firmicutes* were the most frequently reported, identified in both HNSCC patients and healthy controls across eight studies [21,23,26,27,28,29,31,33]. However, their relative abundance varied: five studies reported enrichment in HNSCC patients [26,28,29,31,33], while three found a higher prevalence in healthy controls [21,23,27]. These inconsistencies suggest other potential confounding variables, such as tumour location, disease stage, and host–microbiome interactions, may affect this relationship.

*Actinobacteria*, reported in six studies [6,21,24,27,28,29], were consistently depleted in HNSCC patients in five studies [21,24,27,28,29], with none reporting reductions in controls. This consistent trend suggests a potential protective function in maintaining microbial homeostasis and warrants further functional investigation.

Similarly, *Proteobacteria*, reported in four studies [27,28,29,31], were reduced in HNSCC patients in three studies. One study also reported a decrease in healthy controls. Reduced *Proteobacteria* may reflect a shift toward a pro-inflammatory, anaerobic microbiome, a hallmark of tumorigenesis. Similar trends have been observed in colorectal cancer [41], supporting a broader role for *Proteobacteria* in cancer-associated dysbiosis. *Synergistetes*, identified in one study [21], were enriched in HNSCC patients, suggesting a potential contribution to oral microbial imbalance in cancer.

At the class level, *Bacteroidia* (phylum *Bacteroidetes*) and *Fusobacteriia* (phylum *Fusobacteria*) were consistently enriched in HNSCC patients across six studies, suggesting a strong correlation with-inflammation and tumour-promoting environments [42]. Elevated *Fusobacteriia* have been associated with immune evasion, hypoxia, and tumor progression, while *Bacteroidia* are implicated in mucosal barrier disruption and chronic inflammation [43].

At the genus level, distinct shifts were observed between HNSCC patients and healthy controls. *Fusobacterium*, reported in 12 studies, was consistently enriched in patients, reinforcing its role in epithelial invasion, immune modulation, and inflammation-driven tumor progression [44,45]. In contrast, *Streptococcus*—dominant in healthy controls across 10 studies—may contribute to oral microbial stability, with its depletion indicating dysbiosis [46]. *Actinomyces* and *Rothia*, also reduced in patients, are commensal genera associated with a healthy oral microbiome [47].

Pathogenic genera such as *Prevotella* [29,32,33,34], *Porphyromonas* [32,33,34], and *Parvimonas* [6,18,23,25,29] were more abundant in HNSCC patients, consistent with their known roles in inflammation, immune disruption, and carcinogenic metabolite production. *Corynebacterium*, more prevalent in controls, may support mucosal integrity, whereas *Peptostreptococcus*, enriched in four studies [18,25,29,31], is associated with oncogenic pathways. *Veillonella*, reduced in HNSCC patients [24,26,30,34], may reflect a loss of microbial diversity and ecological stability. Other genera such as *Neisseria*, *Haemophilus*, *Capnocytophaga*, *Gemella*, and *Treponema* showed variable distributions, underscoring the complexity of host–microbiome interactions in HNSCC. A recent study found *Fusobacterium*, *Peptostreptococcus*, and *Prevotella* enriched in gingival squamous cell carcinoma tissues [41], while *Streptococcus*, *Neisseria*, and *Haemophilus* were predominant in normal buccal mucosa [43]. Less frequently reported taxa, including *Bacillus*, *Megasphaera*, *Kingella*, and *Selenomonas* (control group) [17,21,24] and *Eikenella, Granulicatella*, and *Lachnospira* (patient group) [19,24], require further validation due to limited evidence.

Among species-level findings, *Fusobacterium nucleatum* was consistently enriched in HNSCC samples. This Gram-negative anaerobe is implicated in Toll-like receptor signalling, immune suppression, epithelial invasion, and co-aggregation with other pathogens [48,49]. In contrast, *Streptococcus anginosus*, *S. australis*, *S. constellatus*, *S. mitis*, and several *Haemophilus* species (e.g., *H. influenzae*, *H. parainfluenzae*, *H. pittmaniae*, *H. sputorum*) were predominantly found in healthy individuals, suggesting their roles as core members of a protective oral microbiota [6,50]. Notably, *Streptococcus* species can inhibit *F. nucleatum*-induced inflammation [46], and their depletion may exacerbate tumour-associated immune dysregulation [51].

*Prevotella intermedia*, reported in four studies [16,20,26,29], was linked to tumour cell proliferation and production of methyl mercaptan, a compound associated with oxidative stress and DNA damage [52,53]. While *Lactobacillus* spp. are generally considered beneficial [54], they were elevated in patients in two studies, and thus their role in oral carcinogenesis remains unclear. *Porphyromonas gingivalis*, a known periodontal pathogen, was also enriched in HNSCC samples, consistent with its established involvement in immune evasion, inflammation, and cancer progression [40].

Although our primary analysis focused on comparing the oral microbiota of HNSCC patients with that of healthy controls, it is important to acknowledge the potential relevance of paracancerous tissues in understanding cancer-associated microbial shifts [26,29]. Paracancerous tissue, defined as histologically normal or pre-malignant mucosa adjacent to tumor sites, may exhibit distinct microbial profiles that differ from both tumor tissue and healthy mucosa. These altered profiles may reflect early dysbiosis and the development of a pro-inflammatory microenvironment that contributes to malignant transformation. Furthermore, investigating microbial changes in paracancerous regions may enhance our understanding of field cancerization, a process in which widespread epithelial alterations occur beyond the visible tumor margins. Such insights could support the identification of early diagnostic biomarkers and inform microbiome-targeted preventive or therapeutic strategies in the clinical management of HNSCC.

To explain the observed associations between the oral microbiome and HNSCC, two main hypotheses have been proposed [55]. The *bacteria-before-tumor* hypothesis suggests that dysbiosis contributes to carcinogenesis by promoting a pro-inflammatory and immunosuppressive microenvironment, thereby facilitating epithelial damage and malignant transformation. In contrast, the *bacteria-after-tumour* hypothesis posits that microbial shifts are a consequence of tumour formation, where changes in the tumour microenvironment favor bacterial colonisation and persistence [56]. However, it remains unclear whether oral microbiota dysbiosis plays a causative role in head and neck cancer development or emerges as a consequence of tumor-related changes. This ambiguity, commonly referred to as the “before or after tumor” dilemma, poses a significant challenge to interpreting current findings. Moreover, a multitude of confounding factors, including smoking, alcohol use, diet, oral hygiene, and the effects of cancer treatment, further complicate causal inference [57]. While our review does not attempt to establish causality, it synthesizes consistent microbial patterns observed across diverse studies, which may reflect underlying biological processes linked to tumorigenesis. To clarify the temporal and mechanistic relationships between oral dysbiosis and HNSCC, future research should adopt longitudinal designs with rigorous control of confounders, enabling the identification of predictive biomarkers and development of targeted microbiome-based interventions.

Growing evidence also suggests that the oral microbiome may play a critical role in modulating responses to cancer therapies, including radiotherapy [58], chemotherapy [59], and immunotherapy. Dysbiosis of the oral microbiota can influence mucosal immunity and systemic inflammation, potentially affecting treatment efficacy and toxicity profiles. For instance, certain microbial communities may enhance the inflammatory response, thereby exacerbating oral mucositis during radiotherapy, while others may modulate immune checkpoint activity and alter the effectiveness of immunotherapy [60]. Furthermore, microbial metabolites may interfere with drug metabolism, influencing pharmacodynamics and patient outcomes [61]. Although this review did not directly examine treatment response, the distinct microbial signatures identified in HNSCC patients highlight the need for further investigation. Future studies integrating longitudinal microbiome profiling with clinical treatment data are needed to elucidate the prognostic and predictive value of the oral microbiome in head and neck cancer management.

This review has limitations that warrant consideration. First, most included studies utilized 16S rRNA gene sequencing, which restricts taxonomic resolution to the genus level; species-level data were often incomplete or inconsistent. Second, heterogeneity in study populations likely contributed to variability in reported microbiome profiles. Differences in age, sex, lifestyle, tumour site, and tumour stage may all influence oral microbial composition. In particular, the anatomical location of the tumour is known to affect the local microbiome, yet several studies lacked precise tumour site information, which may have introduced inconsistencies. Third, variability in sampling methods, including oral rinses, tissue biopsies, and swabs, likely contributed to differences in microbial profiles. Methodological inconsistencies, such as disparities in DNA extraction protocols, sequencing platforms, bioinformatics pipelines, and statistical analyses, further limit direct comparability across studies. Finally, geographic and cultural factors, along with individual variability and the potential influence of non-local microbiota, may also contribute to the observed discrepancies. To improve reproducibility and enable cross-study comparisons, future research should prioritize methodological standardization, including consistent sampling procedures, sequencing approaches, and analytical frameworks.

Future Directions and Clinical Implications: Although current predictive biomarkers are limited, the identification of microbiome-based diagnostic and prognostic markers could support the development of more personalized treatment strategies for HNSCC. However, further research is needed before the oral microbiome can be reliably implemented in clinical practice. Longitudinal studies that rigorously control for confounding factors and adopt validated sampling protocols will be critical. Specific bacterial taxa, such as *Fusobacterium nucleatum* and *Porphyromonas gingivalis*, which are frequently associated with HNSCC, have emerged as promising candidates for clinical application as non-invasive biomarkers [11,12]. Their increased abundance in patients with HNSCC suggests potential utility in early detection and risk stratification, particularly through the development of molecular assays targeting these organisms. In addition, dynamic profiling of the oral microbiome during treatment, such as chemotherapy, radiotherapy, immunotherapy, or combination therapies, may serve as a surrogate indicator of therapeutic response and treatment-related toxicity, offering opportunities for real-time patient monitoring. Understanding the interactions between these microbial species and the tumour microenvironment also opens new avenues for microbiome-targeted therapies. Strategies to modulate dysbiosis, including the use of probiotics [62], selective antibiotics [63], dietary interventions [64], or bacteriophage therapy, may enhance treatment outcomes when combined with conventional oncologic modalities [65]. Furthermore, integrating oral microbiome data with multi-omics platforms, such as host genomics, transcriptomics, and metabolomics, could enhance the predictive accuracy of disease models and facilitate personalized therapeutic strategies. Collectively, these approaches underscore the translational relevance of oral microbiome research in advancing precision oncology for head and neck cancer.

## 5. Conclusions

In conclusion, our study identified significant shifts in the oral microbiome of HNSCC patients, notably increased *Fusobacterium nucleatum* and *Prevotella intermedia*, and reduced *Streptococcus mitis* and *Rothia mucilaginosa*, indicating a pro-inflammatory, tumor-promoting environment. However, methodological and population heterogeneity limit current conclusions. Standardised, well-controlled studies are needed to determine if there is any causality in the relationship between oral microbiome and HNSCC, and support the development of microbiome-based diagnostic, prognostic, and therapeutic strategies in head and neck cancer.

## Figures and Tables

**Table 1:** Oral microbiota studies in HNSCC.

First Author, Year, Country	Sample Size Age/Range	Treatment/Site		Stage	Lifestyle		Sample Collection	Sample Analysis	Results
					Smoking	Alcohol			
de Freitas Neiva Lessa, A. et al., 2024, Brazil [32]	P (n= 49) C (n = 25) P (59 yrs) C (53 yrs)	CRT (n= 33) SRT (n = 7) SCRT (n = 5) RT alone (n = 4)	Oropharynx cancer (n = 20) Larynx cancer (n = 15) Oral cancer (n = 14)	Oropharynx cancer [stages IVA (n = 12), IVB (n = 6) and III (n = 2)], larynx cancer [stages IVA (n = 8), III (n = 4), I (n = 2), and IVB (n = 1)], oral cancer [stages IVA (n= 12), IVB (n =1), and III (n = 1)]	C (n = 8) P (n = 21)	C (n = 5) P (n = 7)	Oral Swab	16S rRNA V1–V2	*Bacteroidetes* were significantly more abundant in patients with HNSCC, whereas *Firmicutes*—particularly members of the genus Streptococcus—were depleted. Within the *Bacteroidetes* phylum, *Prevotella* and *Porphyromonas* were the predominant genera.
Unlu, O. et al., 2024, Turkey [31]	P (n= 10) C (n= 12) P (61 yrs) C (57 yrs)	Before Treatment	Oral cavity cancer	N/A	P (n = 10) C (n = 8)	P (n = 2) C (n = 4)	Saliva	16S rRNA V3–V4	Patients with oral cancer exhibited poorer oral health and a distinct oral microbiome composition influenced by daily personal habits, which may contribute to disease pathogenesis. Improved oral hygiene and management of periodontal disease may help limit oral cancer development and progression.
Aparna, K. et al., 2024, India [29]	P (n= 13) C (n= 13) P (55 yrs)	N/A		Stage II (pT2N0) Stage III (pT3N0) Stage IVA (pT4aN0)	N/A	N/A	Tumor Tissue, Normal Tissue	16S rRNA V3–V4	*Fusobacterium, Prevotella, Capnocytophaga, Leptotrichia, Peptostreptococcus, Parvimonas,* and members of the *Bacteroidetes* phylum were significantly enriched in oral squamous cell carcinoma (OSCC) lesions compared to adjacent non-cancerous tissues.
Mäkinen, A. et al., 2023, Finland [30]	P (n= 99) C (n= 101) P (68 yrs) C (66 yrs)	RT (n = 44) CT (n = 5)	Oral cavity cancer of squamous cell origin	Stages I–II (n = 57) Stages III–IV (n = 42)	P (n = 43) C (n = 8)	P (n = 62) C (n = 59)	Saliva	16S rRNA V4	Salivary microbial profiles differed significantly between patients with oral squamous cell carcinoma (OSCC) and healthy controls. At baseline, OSCC patients exhibited ecologically adverse alterations—namely, increased proportions of aciduric taxa, reduced α-diversity, and elevated relative abundances of potentially pathogenic taxa.
Lan, Q. et al., 2023, China [24]	P (n= 18) C (n= 21) P (54 yrs) C (48 yrs)	Before Treatment	Oral cavity cancer	N/A	P (n = 5) C (n = 2)	P (n = 5) C (n = 2)	Saliva	Metagenomic sequencing	Salivary microbiota profiles differed significantly among patients with oral squamous cell carcinoma (OSCC), oral leukoplakia (OLK), and healthy controls (HCs). These compositional and functional alterations in the salivary microbiota may be linked to OSCC progression.
Benjamin, W.J. et al., 2023, USA [17]	P (n= 52) C (n= 102) P (59 yrs) C (59 yrs)	Before Treatment	Larynx Oral cavity Oropharynx Hypopharynx Nasal cavity, Sinus, or Skull Unknown primary	Stage 1 or 2 (34%) Stage 3 (17%) Stage 4 (48%)	P {(Never (n = 15) Former (n = 24) Current (n= 2)} C {(Never (n = 51) Former (n = 36) Current (n = 9)}	P {(Never (n = 3) Former (n = 12) Current (n = 26)} C {(Never (n = 5) Former (n = 18) Current (n = 72)}	Oral Wash	16S rRNA V4	Patients with HNSCC exhibited enrichment of the families Lachnospiraceae and Eikenella—taxa previously implicated in periodontitis—suggesting that preservation of a healthy oral microbiome may confer protection against HNSCC. A community type dominated by periodontitis-associated genera (*Fusobacterium* and *Prevotella*) was more frequently observed in older individuals and HNSCC patients, whereas a community enriched in commensal taxa (*Streptococcus* and *Rothia*) was more common in younger, cancer-free controls.
Yan, K. et al., 2023, USA [18]	P (n= 35) C (n= 31) P (66 yrs) C (64 yrs)	Before Treatment	Oral cavity	T_1_-T_2_ (n = 22) T_3_-T_4_ (n = 13)	P:{(Never (n = 13) Former (n = 14) Current (n = 8)} C:{(Never (n = 12) Former (n = 16) Current n = 3)}	P {(Yes (n = 23) No (n = 11)} C {(Yes (n = 15) No (n = 13)}	Oral Swabs	16S rRNA N/A	Alterations in the relative abundance of bacterial genera have been associated with oral cavity carcinogenesis and disease progression. The abundance of several genera—including *Fusobacterium*, *Peptostreptococcus*, *Parvimonas*, *Neisseria*, and *Treponema*—was positively correlated with advancing tumor stage.
Oyeyemi, B.F. et al., 2023, India [28]	P (n= 10) C (n= 10) P (55 yrs) C (23 yrs)	N/A	Oral cavity	Stage I (n = 1) Sage II (n = 1) Stage III (n = 4) Stage IV (n = 4)	P (n = 9) C (n = 0)	P {(Yes (n = 7) No (n = 03)} C {(Yes (n = 00) No (n = 10)}	Saliva	16S rRNA V3–V4	The significant role of dysregulated microbial taxa in the development of oral squamous cell carcinoma (OSCC) and their association with smokeless tobacco use has been identified. Abnormal alterations in the oral microbiota may trigger chronic inflammatory responses, potentially leading to the activation of oncogenes and other tumor-promoting pathways.
Wu, Z. et al., 2023, USA [20]	P (n= 53) C (n= 110) P (72 yrs) C (71 yrs)	N/A	Oral cavity Pharynx	N/A	P {(Never (n = 15) Former (n = 32) Current (n = 06)} C {(Never (n = 29) Former (n = 65) Current (n = 16)}	P {(Yes (n = 46) No (n = 07)} C {(Yes (n = 90) No (n = 20)}	Oral Wash	Shotgun metagenomic sequencing	Alpha and beta diversity did not differ significantly between head and neck cancer (HNC) patients and controls. However, the presence of oral fungi and the relative abundance of several microbial species—including red and orange complex periodontal pathogens—were associated with a reduced risk of HNC.
Pandey, D. et al., 2022, Australia [34]	P (n= 21) C (n= 27) P (59 yrs) C (63 yrs)	Before Treatment	Mucosal squamous carcinoma of the tongue, buccal mucosa, tonsil, palate, hypopharynx, larynx	N/A	P (n = 38.1%) C (n = 22.2%)	P (n = 38.1%) C (n = 38.1%)	Saliva Tissue Oral Swab	16S rRNA V3–V4	Saliva, tissue, and oral swab samples were compared to evaluate their utility in oral microbiome analysis. Saliva microbiomes were found to be the most diverse and exhibited higher temporal stability. Moreover, salivary profiles effectively distinguished HNC patients from healthy controls.
Ganly, I. et al., 2022 USA [21]	P (n= 42) C (n= 45) P (63 yrs) C (63 yrs)	SRT (n = 24) SR post RT (n = 18)	Tongue 24 (57%) Floor of mouth 5 (12%) Upper gum 3 (7.2%) Lower gum 6 (14%) Buccal 2 (4.8%) Retromolar trigone 2 (4.8%) Lip	Stage I (n = 20) Stage II (n = 4) Stage III (n = 6) Stage IV (n = 12)	P {(Never (n = 22) Quit (n = 20)} C {(Never (n = 24) Quit (n = 21)}	P {(Yes (n = 27) No (n= 15)} C {(Yes (n = 30) No (n = 15)}	Oral Wash	Shotgun metagenomic sequencing	The taxonomic composition of the oral microbiome in patients with oral cavity squamous cell carcinoma (OC-SCC) is similarly altered in both smokers and non-smokers.
Mougeot, J.L.C. et al., 2022, USA [22]	P (n= 23) C (n= 20) P (49 yrs) C (66 yrs)	N/A	Base of tongue Nasopharynx Oral cavity Oropharynx Supraglottis Tongue Tonsil	N/A	N/A	N/A	Saliva, Oral Swab	16S rRNA V3–V4	The oral microbiome profiles of HNC patients with HPV-positive (HPV^+^) and HPV-negative (HPV^−^) status differed significantly, particularly in the abundance of periodontal-associated species. The findings suggest that certain oral bacterial species, such as *Leptotrichia* spp., which possess unique ecological niches and invasive capabilities, may coexist with HPV within HPV-induced oral lesions.
Chan, J.Y.K. et al., 2022, China [25]	P (n= 76) C (n= 76) P (≤ 60 = 29,> 60 = 47) C (≤ 60 = 31, >60 = 45)	Pre and Post Surgery	Oral cavity (n = 45) Larynx (n = 12) Oropharynx (n = 11) Hypopharynx (n = 5) Nasal cavity (n = 2) Paranasal sinus (n = 1)	T_1_ (n = 23) T_2_ (n = 20) T_3_ (n = 9) T_4_ (n = 24) N_0_ (n = 40) N_1_ (n = 14) N_2_ (n = 22)	P {(Yss n = 26, No(n= 50) C {(Yes (n= 26), No (n= 50)}	P {(Yes (n = 18), No (n = 58)} C {(Yes (n = 17) No (n = 59)}	Oral Rinse	16S rRNA V3–V4	Oral microbiome dysbiosis associated with HNSCC is dynamic, showing a post-treatment trend toward the re-establishment of microbial communities that resemble those of healthy individuals. These post-treatment microbiome shifts were also associated with patient outcomes and may serve as potential biomarkers for prognosis and clinical management in HNSCC.
Sharma, A.K. et al., 2020, USA [16]	P 27 C 24 P (58 yrs) C (48 yrs)	N/A	Oral cavity (n = 6) Oropharynx (n = 12) Larynx (n = 6) Hypopharynx (n = 2) N/A (n = 1)	Stage I = 3 Stage II = 5 Stage III = 4 Stage IV = 15	C (18.8 mean Per day) Cases (13.9 mean per day)	(Never = Case7, Control 8) (Monthly = Case 2, Control 4) (2-4/month= Case 3, Control 5) (> 4/month = Case13, Control 7) (N/A= Case 2, Control 0)	Brushing Oral Mucosa	16S rRNA V4	Significant alterations in the oral microbiome were observed between smokers with oral and head and neck cancer (HNC) and cancer-free smokers. Specific bacterial taxa—most notably *Stenotrophomonas*—were positively associated with elevated DNA adduct levels and enhanced xenobiotic metabolism. Additionally, increased bacterial richness and diversity, along with the presence of these taxa, were linked to tobacco-related oral and head and neck carcinogenesis.
Zhang, L. et al., 2020, China [26]	P (n = 50) C (n = 50) P (61 yrs) C (61 yrs)	NA	Oral buccal mucosa	Stage I (n = 23) Sage II (n = 16) Stage III (n = 8) Stage IV (n = 3)	P {(Never (n = 24) Former (n = 17) Current (n = 09)}	P {(Never (n = 13) Former (n = 20) Current (n = 17)	Cancer Tissue, Normal Tissue	16S rRNA V3–V4	Oral bacterial profiles showed significant differences between cancer sites and normal tissue of OSCC patients, which might be considered diagnostic markers and treatment targets.
Zhang, Z. et al., 2019, China [27]	P (n= 30) C (n = 30) P (58 yrs) C (58 yrs)	Before Treatment	Cheek Gingiva Oropharynx Tongue Others	Stage I-II (n = 25) Stage III-IV (n = 5)	P {(Yess (n = 08), No (n = 22)}	P {(Yess (n = 07), No (n = 23)}	Tissue Saliva Mouth wash	16S rRNA V1–V2	The microbiota was compared with OSCC tissue, saliva, and mouthwash samples collected from the same subjects. In OSCC tissue, *Acinetobacter* and *Fusobacterium* were the most abundant taxa, particularly in late-stage OSCC. Their known roles in promoting infection and local inflammation suggest a potential contribution to OSCC progression.
Hayes, R.B. et al., 2018, USA [19]	P (n= 129) C (n= 254) P (71 yrs) C (71.0 yrs)	N/A	Oral (n = 41) Pharynx (n = 30) Larynx (n = 58)	N/A	P {(Never (n = 18) Former (n = 70) Current (n = 41)} C {(Never (n = 129) Former (n = 115) Current (n = 10)}	P {(Yess (n = 86), No (n = 20)} C {(Yess (n = 157), No (n = 68)}	Oral Rinse	16S rRNA V3–V4	An increased abundance of *Corynebacterium*, *Kingella*, and other selected genera and species was associated with an elevated risk of HNSCC. This study provides the first comprehensive evidence linking the oral microbiome to the subsequent risk of HNSCC, with the strongest associations observed in laryngeal cancer and among individuals with a history of tobacco use.
Vesty, A. et al., 2018, New Zealand [35]	P (n= 14) C (n= 07) P (49-81yrs) C (20-35 yrs)	N/A	Left parotid Buccal mucosa Right tongue Left palate Floor of mouth Lateral tongue Left tonsil Base of tongue	N/A	P {(Never (n = 05) Former (n = 06) Current (n = 02)} C:(Never (n = 07)	N/A	Saliva	16S rRNA V3–V4	This study reported that the utility of salivary bacterial communities as biomarkers for head and neck squamous cell carcinoma (HNSCC) is limited, due to their reduced capacity to distinguish HNSCC patients from dentally compromised individuals.
Yang, C.-Y. et al., 2018, Taiwan [6]	P (Stage 1, n= 41 Stage 2 and 3, n = 66 Stage 4, n = 90) C (n= 51) P (Stage 1= 53 yrs Stage 2,3 = 54 yrs Stage 4 = 52 yrs) C (31 yrs)	Before Treatment	Buccal mucosa; Tongue; Gingiva; Mouth floor; Others	Stage I (n = 41) Stage II (n = 49) Stage III (n = 17) Stage IV (n = 90)	P {Stage 1= Yes (n = 26), No (n = 15) Stage 2,3 = Yes (n = 50) No (n = 16) Stage 4 = Yes (n = 63), No (n = 27)} C (N/A)	P {(Stage 1= Yes (n = 23), No (n= 18) Stage 2,3 = Yes (n = 36), No (n = 30) Stage 4 = Yes (n = 51), No (n = 39)} C (N/A)	Oral Rinse	16S rRNA V3–V4	The oral microbiota community undergoes dynamic changes during the progression of oral cancer. A bacterial marker panel—characterized by upregulation of *Fusobacterium periodonticum* and downregulation of *Streptococcus mitis* and *Prevotella pasteri*—was able to discriminate stage IV oral squamous cell carcinoma (OSCC) patients from healthy controls.
Lim, Y. et al., 2018 Australia [33]	P (n= (HPV- =21 HPV⁺ =31) C (n= 20) P (HPV-ive >50 =20 yrs HPV⁺ive >50 = 31 yrs)	N/A	Oral cavity Oropharyngeal	HPV- (n = 21) Stage I (n = 3) Stage II (n = 3) Stage III (n = 6) Stage IV (n = 9) HPV⁺ (n = 31) Stage I (n = 1) Stage II (n = 1) Stage III (n = 4) Stage IV (n = 25)	P { HPV-ive (Never (n = 2) Former (n = 16) Current (n = 3) HPV⁺ive (Never (n = 8) Former (n = 30) Current (n = 03)}	P {(HPV-ive Yes (n = 14) No (n = 7) HPV⁺ive Yes (n = 12) No (n = 19)	Oral Rinse	16S rRNA V6–V8	An oral microbiome panel comprising *Rothia*, *Haemophilus*, *Corynebacterium*, *Paludibacter*, *Porphyromonas*, *Oribacterium*, and *Capnocytophaga* effectively distinguished age-matched healthy controls from patients with oral cavity cancer (OCC) and oropharyngeal cancer (OPC) with high accuracy.
Wang, H. et al., 2017 USA [23]	P (n= 121) C (n= 121) P (63 yrs) C (63 yrs)	SUR (n = 21) CT (n = 24) RT (n = 30)	Oral cavity Oropharynx Hypopharynx Larynx	Stage I–II (n = 24) Stage III–IV (n = 78)	P {(Never (n = 29) Former (n = 68) Current (n = 18)}	P {(Never (n= 37) Former (n = 10), Current (n = 67)}	Tumor Tissue, Normal Tissue	16S rRNA V1–V4	The microbiomes of HNSCC tumor microenvironments are largely similar in overall diversity and bacterial composition to those of histologically normal adjacent tissues. However, the study identified a decrease in the abundance of the genus *Actinomyces* and its higher-level taxa up to the phylum level, with this reduction being more pronounced in samples from higher T-stage tumors.

P: patient, C: control, SUR: surgery, CT: chemotherapy, RT: radiotherapy, CRT: chemotherapy and radiotherapy, SRT: surgery and radiotherapy, SCRT: surgery, chemotherapy, and radiotherapy, N/A: not applicable, Yrs: years, n: number.

**Table 2:** Oral microbiota in HNSCC patients versus healthy controls.

First Author, Year, Country	Diversity		Phyla		Class		Genus		Species	
	Alpha	Beta	HNSCC	HC	HNSCC	HC	HNSCC	HC	HNSCC	HC
de Freitas Neiva Lessa, A. et al., 2024, Brazil [32]	S	NS					*Prevotella*↑ *Porphyromonas*↑ *Fusobacterium*↑ *Streptococcus*↓ *Actinomyces*↓ *Leptotrichia*↓ *Corynebacterium*↓ *Rothia*↓	*Prevotella*↓ *Porphyromonas*↓ *Fusobacterium*↓ *Streptococcus*↑ *Actinomyces*↑ *Leptotrichia*↑ *Corynebacterium*↑ *Rothia*↑		
Unlu, O. et al., 2024, Turkey [31]	S	S	*Firmicutes*↑ *Proteobacteria*↓	*Proteobacteria*↑ *Firmicutes*↓			*Streptococcus*↑ *Gemella*↑ *Peptostreptococcus*↑ *Fusobacterium*↑	*Streptococcus*↓ *Gemella*↓ *Peptostreptococcus*↓ *Fusobacterium*↓	*F. nucleatum*↑ *Lactobacillus* spp↑ *Rothia mucilaginosa*↑ *Granulicatella adiacens*↑ *Neisseria elongate*↓ *Aggregatibacter aphrophilus*↓ *Haemophilus sputorum*↓ *Actinomyces massiliensis*↓ *Veillonella* spp↓	*Neisseria elongata*↑ *Aggregatibacter aphrophilus*↑ *Haemophilus sputorum*↑ *Veillonella* spp↑ *Actinomyces massiliensis*↑
Aparna, K. et al., 2024, India [29]	NS	NS	*Firmicutes*↑ *Proteobacteria*↓ *Actinobacteria*↓	*Proteobacteria*↑ *Actinobacteria*↑	*Fusobacteria*↑ *Bacteroidetes*↑	*Fusobacteria*↓ *Bacteroidetes*↓	*Fusobacterium*↑ *Prevotella*↑ *Capnocytophaga*↑ *Leptotrichia*↑ *Peptostreptococcu*↑ *Parvimonas*↑ *Streptococcus*↓ *Haemophilus*↓	*Streptococcus*↑ *Haemophilus*↑ *Fusobacterium*↓ *Prevotella*↓	*Fusobacterium nucleatum*↑ *Prevotella intermedia*↑ *Streptococcus mitis*↓ *Haemophilus influenza*↓	*Streptococcus mitis*↑ *Haemophilus influenza*↑ *Fusobacterium nucleatum*↓ *Prevotella intermedia*↓
Mäkinen, A. et al., 2023, Finland [30]	S	S					*Veillonella*↓ *Actinomyces*↓	*Veillonella*↑ *Actinomyces*↑ *Prevotella*↑	*Streptococcus anginosus*↑ *Abiotrophia defectiva*↑ *Fusobacterium nucleatum*↑ *Streptococcus australis*↓	*Streptococcus anginosus*↓ *Fusobacterium nucleatum*↓
Lan, Q. et al., 2023, China [24]	NS	S	*Actinobacteria*↓				*Gemella*↑ *Lachnospira*↑ *Granulicatella*↑ *Fusobacterium*↑ *Streptococcus*↑ *Corynebacterium*↓ *Veillonella*↓	*Veillonella*↑ *Corynebacterium*↑ *Gemella*↓ *Streptococcus*↓		
Benjamin, W.J. et al., 2023, USA [17]	NS	S					*Fusobacterium*↑ *Eikenella*↑ *Lactobacillus*↓ *Bacillus*↓ *Acinetobacter*↓	*Lactobacillus*↑ *Bacillus*↑ *Acinetobacter*↑ *Fusobacterium*↓		
Yan, K. et al., 2023, USA [18]	NS	S					*Fusobacterium*↑ *Peptostreptococcus*↑ *Neisseria*↑ *Parvimonas*↑ *Treponema*↑ *Streptococcus*↓ *Rothia*↓ *Actinomyces*↓ *Megasphaera*↓	*Streptococcus*↑ *Rothia*↑ *Actinomyces*↑ *Megasphaera*↑ *Fusobacterium*↓ *Peptostreptococcus*↓ *Neisseria*↓		
Oyeyemi, B.F. et al., 2023, India [28]	NS	NS	*Firmicutes*↑ *Actinobacteria*↓ *Proteobacteria*↓		*Bacteroidetes*↑			*Neisseria*↑ *Leptotrichia*↑ *Campylobacter*↓		
Wu, Z. et al., 2023, USA [20]	NS	S					*Corynebacterium*↓		*Fusobacterium nucleatum*↑ *Porphyromonas gingivalis*↑ *Prevotella intermedia*↑ *Prevotella nigrescens*↑ *Red-complex bacteria*↑ *Kingella oralis*↓	*Kingella oralis*↑ *Corynebacterium matruchotii*↑ *Fusobacterium nucleatum*↓ *Porphyromonas gingivalis*↓
Pandey, D. et al., 2022, Australia [34]	S	S					*Fusobacterium*↑ *Prevotella*↑ *Porphyromonas*↑ *Lactobacillus*↑ *Streptococcus*↓ *Veillonella*↓ *Corynebacterium*↓	*Streptococcus*↑ *Veillonella*↑ *Corynebacterium*↑ *Fusobacterium*↓ *Prevotella*↓ *Porphyromonas*↓		
Ganly, I. et al., 2022 USA [21]	NS	S	*Synergistetes*↑ *Actinobacteria*↓ *Firmicutes*↓	*Actinobacteria*↑ *Firmicutes*↑ *Synergistetes*↓	*Bacteroidetes*↑	*Bacteroidetes*↓	*Fusobacterium*↑ *Corynebacterium*↓ *Streptococcus*↓ *Actinomyces*↓ *Cryptobacterium*↓ *Selenomonas*↓	*Corynebacterium*↑ *Streptococcus*↑ *Actinomyces*↑ *Cryptobacterium*↑ *Selenomonas*↑ *Fusobacterium*↓		
Mougeot, J.L.C. et al., 2022, USA [22]		S							*Leptotrichia* spp↑ *Fusobacterium periodonticum*↑ *Haemophilus pittmania*↑ *Alloprevotella tannerae*↑ *Lachnoanaerobaulum orale*↑	*Rothia mucilaginosa*↑ *Haemophilus parainfluenzae*↑
Chan, J.Y.K. et al., 2022, China [25]	S	S					*Fusobacterium*↑ *Peptostreptococcus*↑ *Capnocytophaga*↑ *Parvimonas*↑ *Leptotrichia*↑ *Streptococcus*↓ *Rothia*↓	*Streptococcus*↑ *Rothia*↑ *Fusobacterium*↓ *Peptostreptococcus*↓ *Capnocytophaga*↓		
Sharma, A.K. et al., 2020, USA [16]	S	S							*Fusobacterium nucleatum*↑ *Porphyromonas gingivalis*↑ *Gemella haemolysans*↑ *Lactobacillus* spp↑ *Tannerella forsythia*↑ *Prevotella intermedia*↑ *Neisseria subflava*↓ *Haemophilus parainfluenzae*↓ *Aggregatibacter actinomycetemcomitans*↓ *Veillonella dispar*↓	*Neisseria subflava*↑ *Haemophilus parainfluenzae*↑ *Aggregatibacter actinomycetemcomitans*↑ *Veillonella dispar*↑ *Fusobacterium nucleatum*↓ *Porphyromonas gingivalis*↓ *Tannerella forsythia*↓ *Gemella haemolysans*↓
Zhang, L. et al., 2020, China [26]	NS	NS	*Firmicutes*↑	*Firmicutes*↓	*Fusobacteria*↑ *Bacteroidetes*↑		*Streptococcus*↓ *Veillonella*↓ *Rothia*↓	*Streptococcus*↑ *Veillonella*↑ *Rothia*↑ *Fusobacterium*↓ *Prevotella*↓ *Porphyromonas*↓	*Fusobacterium nucleatum*↑ *Prevotella intermedia*↑ *Peptostreptococcus stomatis*↑	
Zhang, Z. et al., 2019, China [27]	S	S	*Proteobacteria*↑ *Firmicutes*↓ *Actinobacteria*↓	*Firmicutes*↑ *Actinobacteria*↑ *Proteobacteria*↓	*Fusobacteria*↑	*Fusobacteria*↓	*Acinetobacter*↑ *Campylobacter*↑ *Fusobacterium*↑ *Streptococcus*↓ *Rothia*↓	*Streptococcus*↑ *Rothia*↑ *Acinetobacter*↓ *Campylobacter*↓ *Fusobacterium*↓	*Fusobacterium nucleatum*↑ *Acinetobacter baumannii*↑ *Streptococcus mitis*↓ *Rothia mucilaginosa*↓	*Streptococcus mitis*↑ *Rothia mucilaginosa*↑ *Fusobacterium nucleatum*↓ *Acinetobacter baumannii*↓
Hayes, R.B. et al., 2018, USA [19]	NS	S					*Corynebacterium*↓ *Kingella*↓	*Corynebacterium*↑ *Kingella*↑		
Vesty, A. et al., 2018, New Zealand [35]	S	S					*Treponema*↑ *Actinomyces*↓ *Fusobacterium*↓	*Actinomyces*↑ *Fusobacterium*↑ *Treponema*↓	*Candida albicans*↑	
Yang, C.-Y. et al., 2018, Taiwan [6]	S	S	*Actinobacteria*↑		*Fusobacteria*↑ *Bacteroidetes*↑		*Fusobacterium*↑ *Parvimonas*↑ *Streptococcus*↓ *Haemophilus*↓ *Porphyromonas*↓ *Actinomyces*↓	*Streptococcus*↑ *Haemophilus*↑ *Porphyromonas*↑ *Actinomyces*↑ *Fusobacterium*↓	*Streptococcus constellatus*↑ *Haemophilus influenza*↑ *Filifactor alocis*↑	
Lim, Y. et al., 2018 Australia [33]	S	S	*Firmicutes*↑		*Bacteroidetes*↑		*Fusobacterium*↑ *Prevotella*↑ *Porphyromonas*↑ *Streptococcus*↓ *Neisseria*↓	*Streptococcus*↑ *Neisseria*↑ *Fusobacterium*↓ *Prevotella*↓ *Porphyromonas*↓		
Wang, H. et al., 2017 USA [23]	NS	S	*Firmicutes*↓	*Firmicutes*↑			*Parvimonas*↑ *Actinomyces*↓	*Actinomyces*↑ *Parvimonas*↓		

NS: not significant; S: significant.

## Data Availability

Not applicable since this review did not involve new data.
